# The dural angioleiomyoma harbors frequent *GJA4* mutation and a distinct DNA methylation profile

**DOI:** 10.1186/s40478-022-01384-x

**Published:** 2022-05-31

**Authors:** Arnault Tauziède-Espariat, Thibaut Pierre, Michel Wassef, David Castel, Florence Riant, Jacques Grill, Alexandre Roux, Johan Pallud, Edouard Dezamis, Damien Bresson, Sandro Benichi, Thomas Blauwblomme, Djallel Benzohra, Guillaume Gauchotte, Celso Pouget, Sophie Colnat-Coulbois, Karima Mokhtari, Corinne Balleyguier, Frédérique Larousserie, Volodia Dangouloff-Ros, Nathalie Boddaert, Marie-Anne Debily, Lauren Hasty, Marc Polivka, Homa Adle-Biassette, Alice Métais, Emmanuèle Lechapt, Fabrice Chrétien, Felix Sahm, Philipp Sievers, Pascale Varlet, the RENOCLIP-LOC

**Affiliations:** 1 Department of Neuropathology, Sainte-Anne Hospital, 1, Rue Cabanis, 75014 Paris, France; 2 Inserm, UMR 1266, IMA-Brain, Institut de Psychiatrie et Neurosciences de Paris, Paris, France; 3 Department of Radiology, Gustave Roussy, Univ. Paris-Sud, Université Paris-Saclay, 94805 Villejuif, France; 4 Department of Pathology, Lariboisière Hospital, APHP, 75475 Paris, France; 5 U981, Molecular Predictors and New Targets in Oncology, Team Genomics and Oncogenesis of Pediatric Brain Tumors, INSERM, Gustave Roussy, Université Paris-Saclay, 94805 Villejuif, France; 6 Département de Cancérologie de l’Enfant et de l’Adolescent, Gustave Roussy, Université Paris-Saclay, 94805 Villejuif, France; 7 Department of Neurovascular Molecular Genetics, Saint-Louis Hospital, APHP, 75010 Paris, France; 8 Department of Neurosurgery, Sainte-Anne Hospital, 75014 Paris, France; 9 Department of Neurosurgery, Henri Mondor Hospital, 94000 Créteil, France; 10 Department of Pediatric Neurosurgery, Necker Hospital, APHP, Université Paris Descartes, Sorbonne Paris Cité, Paris, France; 11 Department of Neuroradiology, Sainte-Anne Hospital, 75014 Paris, France; 12 Department of Pathology, CHRU, Nancy, France; 13 Department of Neurosurgery, CHRU, Nancy, France; 14 Service de Neuropathologie, Institut du Cerveau - Paris Brain Institute - ICM, Inserm, CNRS, Hôpitaux Universitaires La Pitié Salpêtrière - Charles Foix, Sorbonne Université, AP-HP, 75013 Paris, France; 15 Department of Pathology, Cochin Hospital, AP-HP Paris, Université de Paris, Paris, France; 16 Paediatric Radiology Department, Institut Imagine INSERM U1163 and U1299, AP-HP, Hôpital Necker Enfants Malades, Université Paris Cité, 75015 Paris, France; 17 Department of Pathology, Hôpital Henri-Mondor, INSERM U955, Université Paris-Est, Créteil, France; 18 Department of Neuropathology, Institute of Pathology, University Hospital Heidelberg, Heidelberg, Germany; 19 Clinical Cooperation Unit Neuropathology, German Consortium for Translational Cancer Research (DKTK), German Cancer Research Center (DKFZ), Heidelberg, Germany

**Keywords:** Dural angioleiomyoma, GJA4, DNA methylation profile

## Abstract

The International Society for the Study of Vascular Anomalies (ISSVA) has defined four vascular lesions in the central nervous system (CNS): arteriovenous malformations, cavernous angiomas (also known as cerebral cavernous malformations), venous malformations, and telangiectasias. From a retrospective central radiological and histopathological review of 202 CNS vascular lesions, we identified three cases of unclassified vascular lesions. Interestingly, they shared the same radiological and histopathological features evoking the cavernous subtype of angioleiomyomas described in the soft tissue. We grouped them together with four additional similar cases from our clinicopathological network and performed combined molecular analyses. In addition, cases were compared with a cohort of 5 soft tissue angioleiomyomas. Three out 6 CNS lesions presented the same p.Gly41Cys *GJA4* mutation recently reported in hepatic hemangiomas and cutaneous venous malformations and found in 4/5 soft tissue angioleiomyomas of our cohort with available data. Most DNA methylation profiles were not classifiable using the CNS brain tumor (version 12.5), and sarcoma (version 12.2) classifiers. However, using unsupervised t-SNE analysis and hierarchical clustering analysis, 5 of the 6 lesions grouped together and formed a distinct epigenetic group, separated from the clusters of soft tissue angioleiomyomas, other vascular tumors, inflammatory myofibroblastic tumors and meningiomas. Our extensive literature review identified several cases similar to these lesions, with a wide variety of denominations. Based on radiological and histomolecular findings, we suggest the new terminology of “dural angioleiomyomas” (DALM) to designate these lesions characterized by a distinct DNA methylation pattern and frequent *GJA4* mutations.

## Introduction

Intracranial vascular lesions encompass a broad spectrum of entities which differ in hemodynamic physiology, structure and prognosis [1–3]. They have been increasingly seen in clinical practice primarily because of new developments in imaging technology [4]. In recent years, an effort has been made to categorize these vascular anomalies, classified over the years in a variety of ways by many authors, often on the basis of blood flow patterns, amplified magnetic resonance imaging (MRI), angiography, histopathological features or demographics [5]. In 1996, the International Society for the Study of Vascular Anomalies (ISSVA) developed a classification scheme with two main groups: proliferative vascular lesions (vascular tumors) and vascular malformations. In 2014, the ISSVA refined its classification system, in order to establish a consistent terminology to serve as a guide for all specialized medical personal: clinicians, radiologists, pathologists and to improve patient management and treatment options [6, 7]. The four main categories of commonly encountered vascular malformations found in the Central Nervous System (CNS) are, as described by McCormick et al. [8], arteriovenous malformations, cavernous angiomas (also known as cerebral cavernous malformations (CCM)), venous malformations, and telangiectasias [9, 10]. In addition, mixed malformations occasionally occur [11, 12]. Molecular alterations are now well-characterized in association with those lesions: *CCM1/2/3, MAP3K3* and *PIK3CA* gene mutations in CNS cavernomas [13–19], and *KRAS* mutations in arteriovenous brain malformations [20]. Moreover, recent studies have shown that using DNA-methylation classification, most histopathological CNS and sarcoma tumors cluster into corresponding methylation classes and are stratified into clinically relevant, molecularly distinct subgroups [21, 22]. Here, we introduce a novel CNS tumor type with recurrent *GJA4* mutation and a distinct DNA-methylation profile, for which we suggest the term ‘dural angioleiomyomas’ (DALM), which is not currently included in the World Health Organization (WHO) Classification of CNS tumors [23].

## Materials and methods

### Cohort

**Fig. 1:**
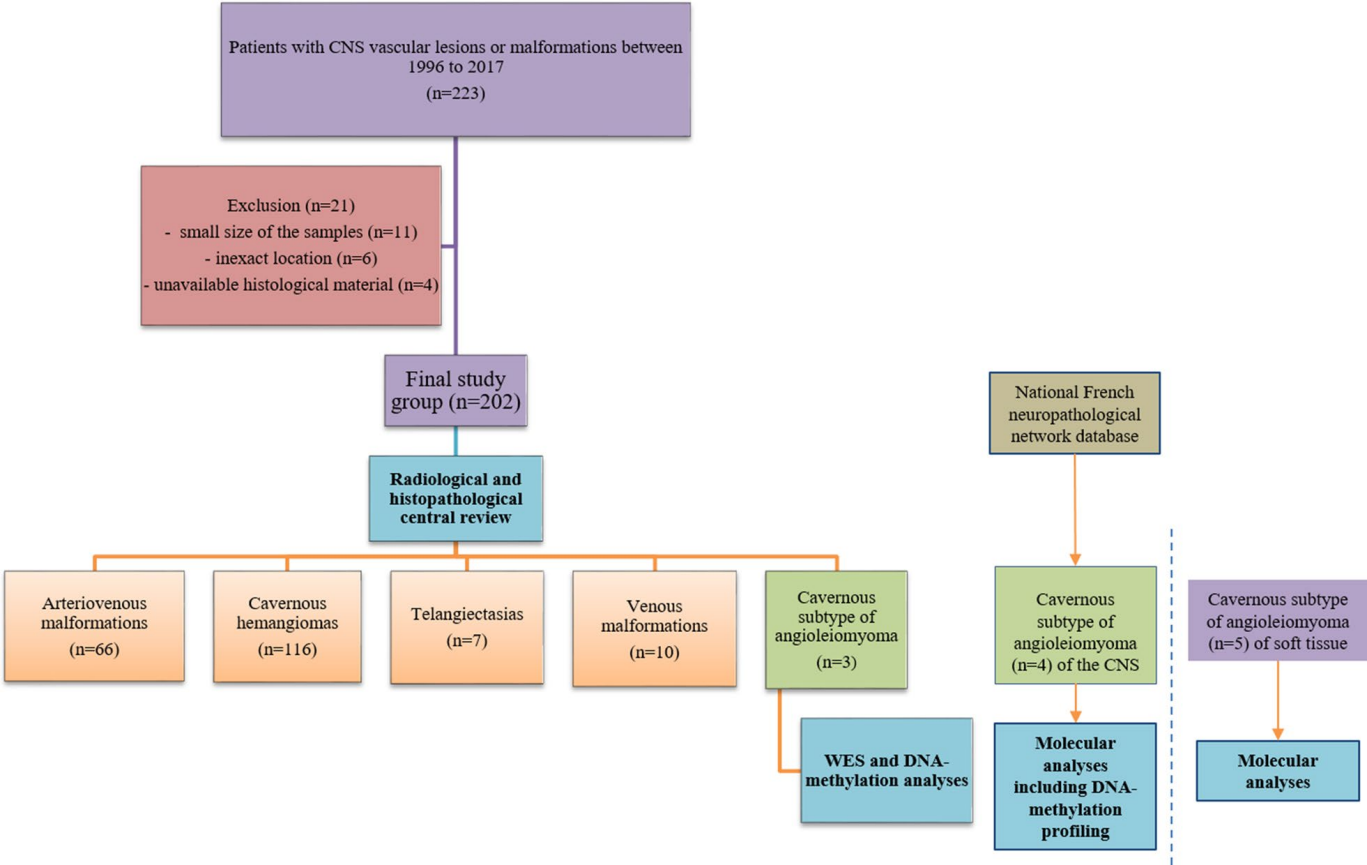
Flowchart of the study. *CNS* Central nervous system

The database from the neuropathological department at Sainte-Anne Hospital in Paris was retrospectively searched for CNS vascular lesions, referred to in reports as vascular lesions, vascular malformations, cavernomas, angiomas, arteriovenous lesions/malformations, or telangiectasias between 1996 and 2017 (Fig. 1). Two hundred and twenty-three cases were retrieved and reviewed by two pathologists (PV and ATE). Twenty-one cases were excluded based on: small sample size (n = 11), an extracranial location (n = 6) or the unavailability of paraffin-embedded sections for additional techniques (n = 4). Among the 202 remaining CNS vascular lesions, 171 were classified into the four ISSVA categories: CCMs (n = 116), arteriovenous malformations (n = 66), venous malformations (n = 10), and telangiectasias (n = 7). Lastly, three lesions remained unclassified which presented similar clinical, imaging, and histopathological features evoking the cavernous subtype of angioleiomyoma. Thereafter, we screened our national French neuropathological network database and found four other cases presenting the same histopathology (one of them was previously described in [24]). We then compared histopathological and molecular features from our 7 CNS cases with a cohort of 5 soft tissue angioleiomyomas.

This study was approved by our local ethics committee. Written informed consent for clinical and imaging investigations and molecular analysis was obtained from all patients enrolled in this study.

### Central radiological review

The central radiological review of preoperative MRI and computed tomodensitometry (CT), when available, was performed by a senior neuroradiologist (TP).

### Central histopathological review and immunohistochemistry

The central pathology review was performed conjointly by 3 neuropathologists (ATE, PV, EL), a pathologist expert in soft tissue tumors (FL), and a pathologist expert in vascular lesions (MW). For the CNS cases, additional immunohistochemical stainings were performed on paraffin-embedded sections including: CD34 (1:40, clone QBEnd10, Dako, Glostrup, Denmark), smooth muscle actin (SMA) (1:1500, clone 1A4, Dako, Glostrup, Denmark), Desmin (1:400, clone D33, Dako, Glostrup, Denmark), h-caldesmon (1:100, clone h-CALD, Santa Cruz Biotechnology, Dallas, USA), PS100 (1:6000, polyclonal, Dako, Glostrup, Denmark), GFAP (1:200, clone 6F2, Dako, Glostrup, Denmark), neurofilament (1:25, clone 2F11, Dako, Glostrup, Denmark), STAT6 (1:200, clone YE361, Abcam, Cambridge, UK), SSTR2a (1:200, clone UMB1, Abcam, Cambridge, UK) and EMA (1:200, clone GM008, Dako, Glostrup, Denmark). All immunohistochemical stainings were performed in an automatic closed immunostainer (Omnis automate). Orcein staining was also performed.

### Molecular analyses

Blood samples were drawn and genomic DNA was extracted using the WIZARD Genomic DNA Purification Kit (Promega, Madison, USA) according to the manufacturer’s protocol for whole blood. DNA was extracted from the tissues by the WIZARD Genomic DNA Purification Kit. When available, the frozen samples were homogenized in a lysis solution, incubated 1 h at 55 °C with proteinase K and DNA isolation was conducted according to the manufacturer’s protocol for Tissue DNA. Sequencing was done with a custom SureSelect^XT^ DNA target enrichment panel designed with SureDesign tools (Agilent Technologies, Santa Clara, USA). All coding and non-coding exons from the three *CCM* genes, and 50 base pairs of intronic flanking sequence were tested both on DNA extracted from peripheral leucocyte and on DNA extracted from frozen tissues. The libraries were prepared according to the SureSelect QXT target enrichment protocol (Agilent Technologies). Sequencing was performed on a MiSeq next generation sequencer (Illumina, San Diego, USA). Alignment of raw data and variant calling and CNV detection was performed using the SeqPilot SeqNext software version 4.0 (JSI Medical Systems). Whole exome sequencing (WES) and bioinformatic analyses were also performed when cryopreserved tissue was available. Library preparation, exome capture, sequencing and raw data analysis was performed by IntegraGen SA (Evry, France). WES was performed on genomic DNA, and matched constitutional DNA extracted from blood samples at IntegraGen (Evry, France). Libraries were prepared from 150 ng of fragmented genomic DNA using the NEBNext Ultra DNA Library Prep Kit for Illumina (New-England Biolabs) and sequences captured using the SureSelect XT Human All Exon CRE-V2 kit (Agilent) followed by paired-end 75 bases massively parallel sequencing on Illumina NextSeq 500. Following base calling using the Real-Time Analysis (RTA2) software sequence pipeline, sequence reads were mapped to the human genome build (hg19) using the Burrows-Wheeler Aligner (BWA) tool. Duplicate readings were removed and variant calling allowing for the identification of genetic alterations as well as SNV (Single Nucleotide Variation) small insertions/deletions (up to 20 bp) were performed via the Broad Institute’s GATK Haplotype Caller GVCF tool (3.7) for constitutional DNA and via the Broad Institute’s MuTect tool (2.0, -max_alt_alleles_in_normal_count = 2; -max_alt_allele_in_normal_fraction = 0.04) for somatic DNA. An in-house post-processing in order to filter out candidate somatic mutations that are more consistent with artifacts or germline mutations was applied. Ensembl’s VEP (Variant Effect Predictor, release 90, GENCODE 27) program was used to process variants for further annotation. Taking into account data available from the dbSNP (dbSNP150), the 1000 Genomes Project (1000G_phase3), the Exome Variant Server (ESP6500SI-V2-SSA137), and the Exome Aggregation Consortium (ExAC r3.0) and in-house databases. Regarding missense changes, two bioinformatics predictions for pathogenicity were available: SIFT (SIFT5.2.2), PolyPhen (2.2.2). To investigate genomic copy number aberrations (CNA), the Bioconductor DNACopy package (DNAcopy 1.32.0) was used by comparing the normal DNA exome data to a reference sample pool.

### DNA methylation array processing and copy number profiling

Genomic DNA was extracted from formalin-fixed and paraffin-embedded (FFPE) tissue of the three undetermined vascular lesions. DNA methylation profiling of all samples was performed using the Infinium MethylationEPIC (850 k) BeadChip (Illumina, San Diego, CA, USA) or Infinium HumanMethylation450 (450 k) BeadChip array (Illumina) as previously described [21]. All computational analyses were performed in R version 3.3.1 (R Development Core Team, 2016; https://www.R-project.org). Copy-number variation analyses from 450 k and EPIC methylation array data was performed using the conumee Bioconductor package version 1.12.0. Raw signal intensities were obtained from IDAT-files using the minfi Bioconductor package version 1.21.4 [21]. Illumina EPIC samples and 450 k samples were merged to a combined data set by selecting the intersection of probes present on both arrays (combineArrays function, minfi). Each sample was individually normalized by performing a background correction (shifting of the 5% percentile of negative control probe intensities to 0) and a dye-bias correction (scaling of the mean of normalization control probe intensities to 10,000) for both color channels. Subsequently, a correction for the type of material tissue (FFPE/frozen) and array type (450 k/EPIC) was performed by fitting univariable, linear models to the log2-transformed intensity values (removeBatchEffect function, limma package version 3.30.11). The methylated and unmethylated signals were corrected individually. Beta-values were calculated from the retransformed intensities using an offset of 100 (as recommended by Illumina). All samples were checked for duplicates by pairwise correlation of the genotyping probes on the 450 k/850 k array. To perform unsupervised non-linear dimension reduction, the remaining probes after standard filtering [21] were used to calculate the 1-variance weighted Pearson correlation between samples. The resulting distance matrix was used as input for t-SNE analysis (t-distributed stochastic neighbor embedding; Rtsne package version 0.13). The following non-default parameters were applied: theta = 0, pca = F, max_iter = 30,000 perplexity = 10.

### Literature review

We performed an extensive review of cases from the English-language literature using the keywords “cavernoma and meninge”, “cavernoma and dura”, “dural cavernoma”, “cavernoma and extraaxial”, “angioma and meninge”, “angioma and dura”, “dural angioma”, “angioma and extraaxial”, “hemangioma and meningeal”, “hemangioma and dura”, “dural hemangioma”, “hemangioma and extraaxial”, “angioleiomyoma and meningeal”, “angioleiomyoma and dura”, “dural angioleiomyoma”, “angioleiomyoma and extraaxial”, “myopericytoma and meningeal”, “myopericytoma and dura”, “dural myopericytoma”, “myopericytoma and extraaxial” in Pubmed.

## Results

### Clinical and imaging findings

**Fig. 2:**
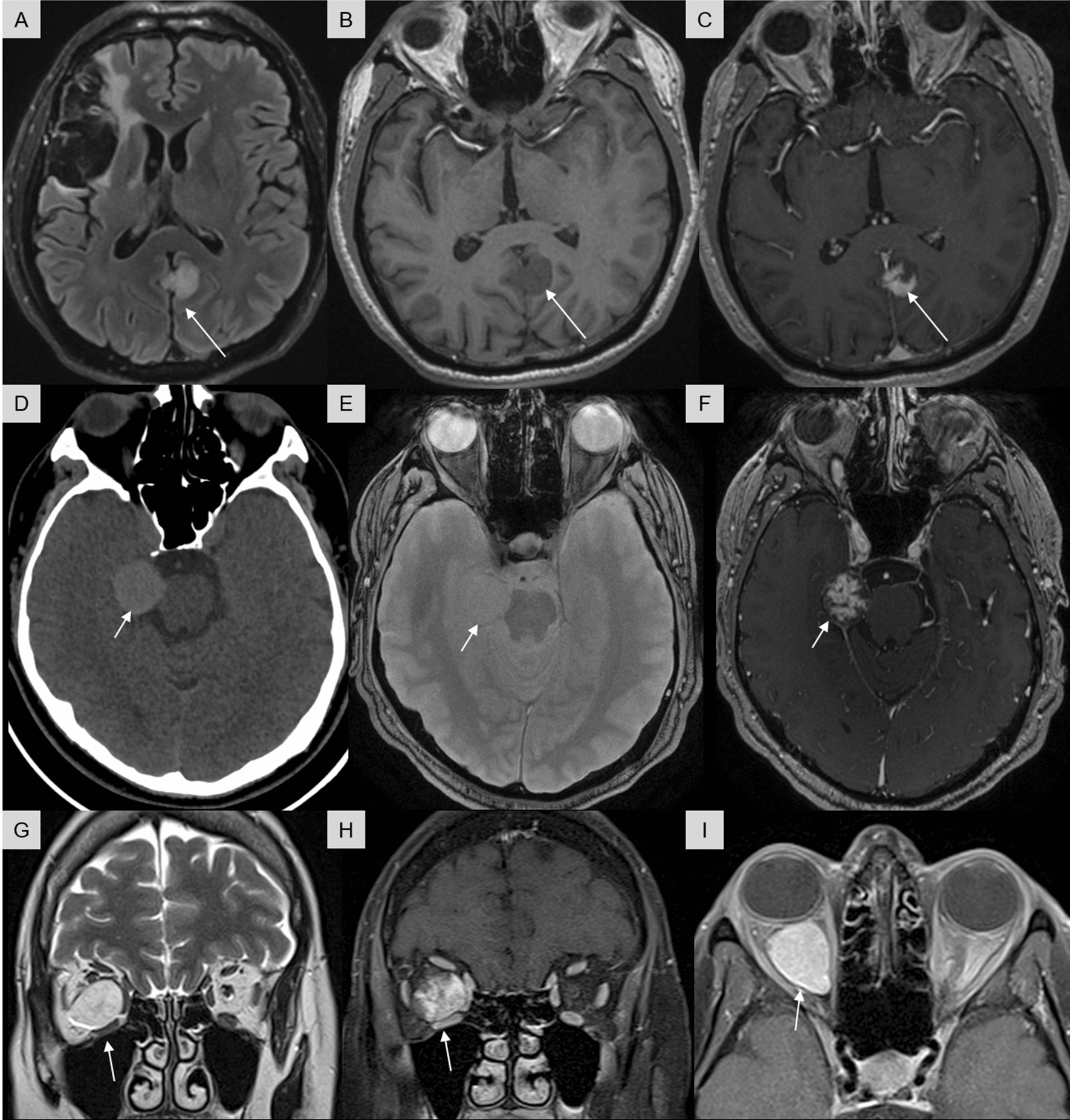
Radiological findings of the three cases of our cohort. Each line represents one patient (cases #1 to #3 from top to bottom). (**A**) Axial Flair sequence showing a homogeneous hyperintense extra-axial mass, well-circumscribed, in the left parietal hemisphere at the level of the posterior third of the falx, without a surrounding parenchymal edema, and the lesion of the right superficial middle cerebral artery territory infarction. (**B**) Axial T1-weighted MR image demonstrating a slightly homogeneous hypointense extra-axial mass. (**C**) After administration of gadolinium, MR axial T1FS image shows a marked and heterogeneous enhancement. Absence of dural tail sign. (**D**) Axial computed tomographic scan showing a round, well-circumscribed, hyperdense extra-axial lesion in the right temporal hemisphere at the level of the anterior third of the cerebellar tentorium. No adjacent edema or significant mass effect. (**E**) Axial T2* sequence showing no haemorrhage or hemosiderin deposits. (**F**) After administration of gadolinium, MR axial T1FS image shows a marked and heterogeneous enhancement. Absence of dural tail sign. (**G**) MRI coronal T2 sequence showing a right intraorbital intraconal mass, well-circumscribed, encapsulated, hyperintense in T2 compared to muscles, lying close to the medial and inferior rectus muscles, abuting the globe, pushing the optic nerve and in contact with the dura mater of the optic nerve. (**H**) After administration of gadolinium, MR coronal T1FS sequence shows a slow gradual and irregular enhancement. (**I**) On delayed contrast enhanced-image, we can notice a full filling of the mass

The clinical data concerning the 7 patients with undetermined vascular lesions are detailed in Table 1. The median age of presentation in our cohort was 52 years (ranging from 46–59). The male-to-female ratio was 1.3 (4 males and 3 females). All tumors were extra-parenchymal, mostly supratentorial (4/7 cases) and three cases were intraorbital (developed from the dura mater of the optic nerve) as confirmed by surgical findings. Data from treatment and follow-up were available for all patients (for details see Table 1). A gross total resection was achieved for all patients with no residual disease. All patients were free of disease at the end of follow-up (mean, 86 months; median, 58 months) including one having survived more than 20 years after resection. Preoperative imaging was available for 6/7 patients. Using neuroimaging, all lesions presented the same features on preoperative MRI: solitary extra-axial, well-circumscribed, attached to the dura mater, and located near the left parietal hemisphere (case #1) (Fig. 2A–C), the right temporal hemisphere (case #2) (Fig. 2D–F), the right cavernous sinus (case #5) the right occipital hemisphere (case #6) and the intraorbital portion of the optic nerve (cases #3, 4, and 7) (Fig. 2G–I). Axial CT scan without a contrast medium was available for only one patient (case #2) and showed a round, well-circumscribed, hyperdense mass with regular margins arising from the cranial dura mater in the right temporal hemisphere (Fig. 2D). No change in the adjacent skull, dural calcification or intralesional calcification was noted (Fig. 2D). They measured in their largest axial diameter between 16 mm (case #1) and 27 mm (case #3) (median: 25 mm, mean: 22.8 mm). The lesions appeared as isointense relative to grey matter on unenhanced T1-weighted MRI and hyperintense on FLAIR and T2-weighted images. FLAIR sequencing revealed an absence of parenchymal edema for intracerebral lesions. Fat-suppressed contrast-enhanced T1-weighted MRI demonstrated an intense and irregular inhomogeneous enhancement after gadolinium administration (Fig. 2C,F,H). No dural tail or bone abnormalities (bony erosion, thinning, or hyperostosis) were noted. No characteristic perilesional hemosiderin deposition, low signal rim or concurrent vascular malformation was observed (Fig. 2E). For the three intraorbital cases, the masses were intraconal, well-circumscribed, encapsulated, hyperintense in T2 compared to muscles, lying close to the rectus muscles, abutting the globe or located in the orbital apex, pushing the optic nerve and in contact with the dura mater of the optic nerve (Fig. 2G–I). No angiographic study was performed for any of the patients because the lesions were misdiagnosed as atypical meningiomas or other dural-based lesions on presurgical MRI. After surgery, imaging confirmed total resection of the lesions.

**Table 1:** Summary of clinical characteristics of cases of our series

Case	Age (y), sex	Site of the tumor	Treatments	Recurrence, PFS (m)	Status at the end of follow-up, OS (m)
1	59, M	Extra-axial (left parietal)	GTR	0	Alive, 58
2	46, M	Extra-axial (right temporal)	GTR	0	Alive, 57
3	59, F	Orbit (optic nerve dura mater)	GTR	0	Alive, 28
4	56, F	Orbit (optic nerve dura mater)	GTR	0	Alive, 97
5	51, M	Cavernous sinus	GTR	0	Alive, 253
6	52, M	Extra-axial (right occipital)	GTR	0	Alive, 109
7	46, F	Orbit (optic nerve dura mater)	GTR	0	Alive, 4

*F* Female, *GTR* Gross total resection, *m* Months, *M* Male, *Y* Years-old

### Histopathological and immunohistochemical characterization

**Fig. 3:**
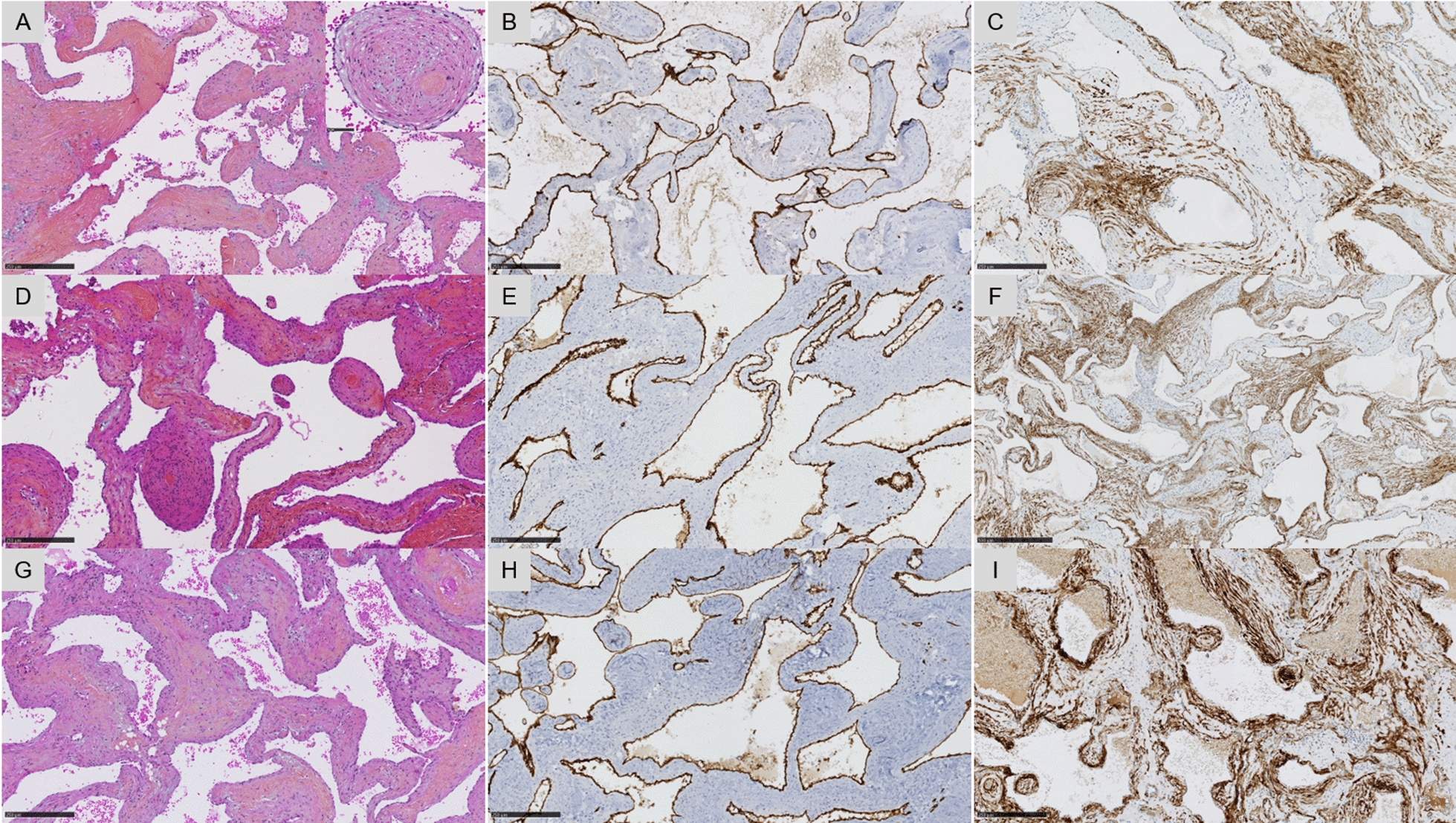
Histopathological features of the three cases of our cohort. Each line represents one patient (cases #1 to #3 from top to bottom). Cavernous-type pattern composed of dilated vascular channels with variable thickening of the walls (**A**, **D**, **G**, HPS, magnification × 100) with some perivascular concentric arrangement of myoid cells (insert magnification × 400). The vascular cavities were lined by endothelial cells stained by CD34 (**B**, **E**, **H**, magnification × 100). In the walls of vascular structure, the tumor cells were diffusely immunoreactive for h-caldesmon (**C**, **F** and **I**, magnification × 100). Black scale bars represent 250 μm (**A**–**I**) and 50 µm (insert). *HPS* Hematoxylin Phloxin Saffron

The seven CNS lesions had the same histopathological features (Fig. 3). They consisted of aggregates of abnormally enlarged vascular cavities (Fig. 3A,D,G), separated by thick uneven fibrous septa and lined by a single layer of endothelial cells stained by the CD34 antibody (Fig. 3B,E,H). There was no intervening brain parenchyma or identifiable mature vessel wall structures. There were not any recent or organized thrombi in the vascular lumens. There were no hemosiderin deposits peripheral to the lesion. The endothelial cells did not present atypia, mitotic activity, or plump epithelioid cytology. We did not identify any elastic lamina by orcein staining. There was no lymphocytic infiltration or calcification. All cases presented myxoid changes. Immunostainings for SMA and h-caldesmon (Fig. 3C,F,I) showed muscular layers of varying thickness surrounding the vascular cavities, including in perivascular concentric arrangements, whereas desmin was only focally expressed. We did not identify nervous fibers or brain parenchyma with PS100 and GFAP immunostainings. These lesions lacked SSTR2a (apart from the endothelial layer), and EMA immunopositivities which are the classical markers of meningioma. STAT6 was not expressed in any of the cases.

### *GJA4* mutation is a frequent event in dural angioleiomyoma

To gain insight concerning the genomic abnormalities underlying these lesion, targeted sequencing for *CCM1/2/3* was performed. The whole genes were fully covered. The mean coverage depth of the targeted regions in panel sequencing data for blood DNA was between 770 and 830X and the mean coverage for tissue DNA was over 6000X. No variant was identified in the exons and flanking introns nor in the blood DNA or the tissue DNA for all three patients. There was no evidence of a large rearrangement of the *CCM1/2/3* genes or of *MAP3K3, PIK3CA* or *KRAS*. Of the seven patients, only two had lesional frozen tissue samples available allowing for a genetic evaluation. WES was performed on tumor genomic DNA (gDNA) and corresponding constitutional gDNA was extracted from blood. The bioinformatic analysis identified 34 and 26 somatic coding single nucleotide variants (cSNV) in the tumors. An identical variant in *GJA4* (NM_002060.3 c.121G > T; p.Gly41Cys) was identified for the two patients. The substitution was reported as *probably damaging* by PolyPhen-2 (score = 1), and accordingly as *damaging* in SIFT prediction (score = 0). For the other cases, we tried to detect this mutation using targeted Sanger sequencing after PCR amplification of the locus on DNA extracted from fixed-formalin paraffin embedded tissues. One of the four tested cases presented the mutation, with valid positive controls. Thereafter, we searched the same mutation by Sanger sequencing in a series of 5 soft tissue angioleiomyomas. All cases tested (4/5) presented the same mutation *GJA4* (p.Gly41Cys), for the last case, the technique was not contributive due to insufficient DNA quality.

### DNA methylation profiling suggest a distinct epigenetic profile

**Fig. 4:**
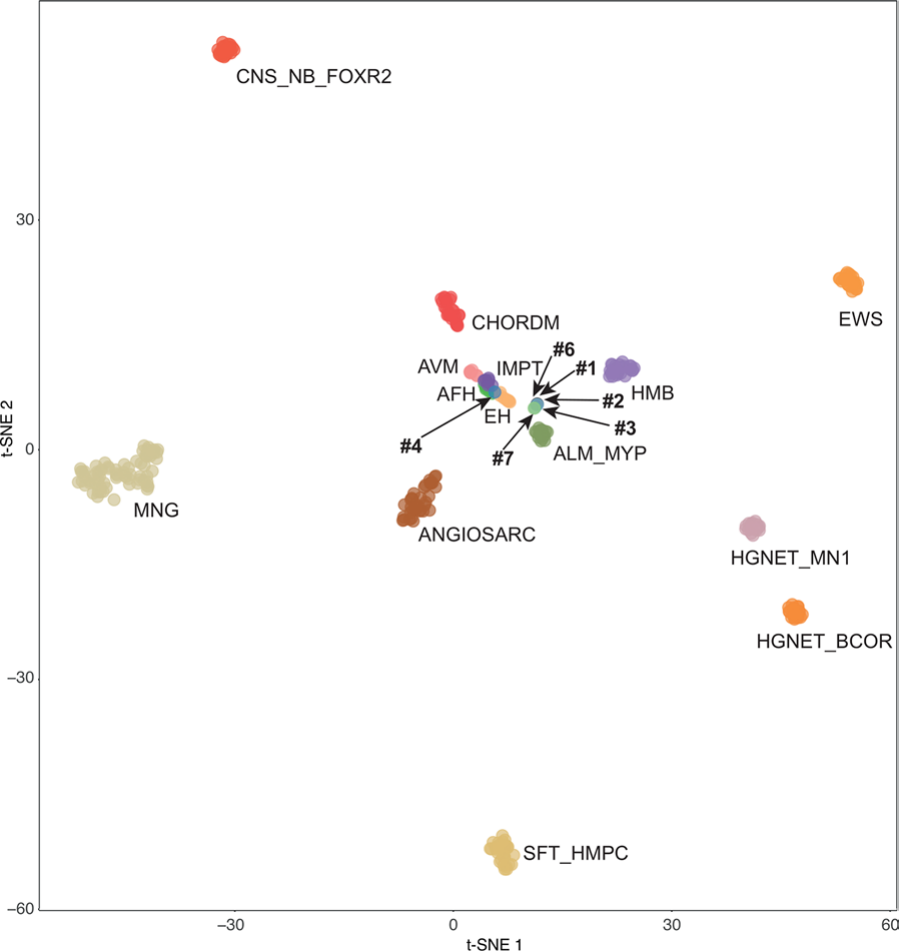
t-distributed stochastic neighbor embedding (t-SNE) analysis of DNA methylation profiles of the six investigated tumors alongside selected reference samples. Reference DNA methylation classes: angiomatoid fibrous histiocytoma (AFH), angioleiomyoma/myopericytoma (ALM_MYP), angiosarcoma (ANGIOSARC), arteriovenous malformation (AVM), chordoma (CHORDM), central nervous system neuroblastoma, *FOXR2*-activated (CNS_NB_FOXR2), epithelioid hemangioendothelioma (EH), Ewing sarcoma (EWS), high grade neuroepithelial tumor with *BCOR* alteration (HGNET_BCOR), high grade neuroepithelial tumor, with *MN1* alteration (HGNET_MN1), hemangioblastoma (HMB), inflammatory myofibroblastic tumor (IMPT), meningioma (MNG), and solitary fibrous tumor/hemangiopericytoma (SFT_HMPC). The cases #1, 2, 3, 6, and 7 clustered together and were separated from other DNA methylation classes

Using DNA methylation-based classification and the Brain Tumor and Sarcoma Classifiers (version 12.5/12.2; www.molecularneuropathology.org), only 1/7 tumor was classifiable (calibrated scores for DNA methylation class > 0.9) (see details in Table 2). Next, a t-Distributed Stochastic Neighbor Embedding (t-SNE) analysis was performed alongside the genome-wide DNA methylation profiles from the sarcoma reference cohort [22] as well as a more focused analysis with selected reference groups. Five of the six cases grouped together and showed no obvious relation to any of the other reference classes such as angioleiomyomas/myopericytomas of the soft tissue, angiosarcomas, epithelioid hemangioendotheliomas, solitary fibrous tumors/hemangiopericytomas, inflammatory myofibroblastic tumors or meningiomas (Fig. 4). No significant copy number alterations were observed in our cases on the copy number variation profiles. The MGMT promotor was unmethylated in all cases and there was a global hypomethylation.

**Table 2:** Summary of molecular characteristics of cases of our series

Case	MC of the sarcoma classifier v12.2 (calibrated score)	MC of the brain classifier v12.5 (calibrated score)	*GJA4* status (variant allele frequency)
1	No match	Meningioma subtype benign subclass 3 (0.87)	pGly41Cys mutation (8%)
2	No match	Meningioma subtype benign subclass 3 (0.47)	pGly41Cys mutation (10%)
3	Lipoma (0,33)	Plexiform neurofibroma (0.46)	pGly41Cys mutation (14%)
4	Angiomatoid fibrous histiocytoma (0.99)	Chordoma (0.30)	WT
5	NA	NA	NA
6	Angioleiomyoma/myopericytoma (0.30)	Meningioma subtype benign subclass 3 (0.78)	WT
7	Well/dedifferentiated liposarcoma (0.41)	Plexiform neurofibroma (0.49)	WT

*MC* Methylation class, *NA* Not available, *WT* Wildype

### Literature review

Our extensive review of cases from the English-language literature, describing only cases with similar radiological and histopathological features to our cases, found 74 similar radiologically and histologically described cases of DALM [24–71]. The median age of patients was 46 (5–79) [26–29], with only one pediatric case [61]. The male/female ratio was 1.4 (43 males and 31 females). The symptoms depended on tumor location, but in 10% of cases the tumors were incidental. Different tumor locations were described, but most of cases were found in the cavernous sinus (43% of cases) and were radiologically interpreted as meningiomas (78% of cases). After resection, only one case recurred (192 months after the initial surgery) [44] and all patients were alive at the end of follow-up. No genetic or epigenetic characterizations were available for any of these cases.

## Discussion

Vascular lesions of the CNS are frequent and represented by intraparenchymal CCM, arteriovenous malformations, venous malformations and telangiectasias. Here, we identified a putative new tumor type characterized by the same dural location, a similar histopathological pattern and a frequent *GJA4* p.Gly41Cys mutation (3/6 cases), distinct from genes implicated in CCM (*CCM1/2/3, MAP3K3, PIK3CA*) and arteriovenous malformations (*KRAS*) of the brain. Particularly, *GJA4* mutations were not found using WES in cavernous malformation cohorts [19]. The *GJA4* gene encodes the Gap Junction Protein Alpha 4 (or Connexin37), a protein from the gap junctions of endothelial cells [72]. Interestingly, a previous study evidenced that connexin37 knockout mice displayed severe vascular abnormalities (particularly in the testis and intestine), looking like “cavernous hemangiomas” and died perinatally from haemorrhage [73]. More recently, the same *GJA4* (p.Gly41Cys) mutation was reported in a subset (as in our work) of a series of vascular lesions located in the liver and the skin [74]. In this study, the vascular lesions were denominated as “hepatic hemangiomas” and “cutaneous venous malformations” but the histopathological data are limited, impeding us from performing a detailed comparison [74]. This recent study showed that this mutation leads to a non-canonic activation of SGK1 (serum/glucocorticoid-regulated kinase 1) which is implicated in various neoplasms (as breast cancer, hepatocellular carcinoma, glioblastoma, colorectal cancer and non-small cell lung cancer) [75–79]. During embryogenesis, SGK1 is necessary for normal angiogenesis with a prosurvival role in endothelial and vascular smooth muscle cells [80]. In our study, we evidenced that dural cases shared morphological similarities with the cavernous variant of angioleiomyomas. They presented well-differentiated smooth muscle cells stained by h-caldesmon and actin with perivascular concentric arrangements of myoid cells intervening dilated vascular channels of variable thickness [81, 82]. The presence of myoid cells arranged circumferentially in layers around the vascular lumina and the absence of expression of desmin may also evoke the diagnosis of myopericytoma, but those features are not specific to this tumor and may be encountered in a subset of angioleiomyomas [82]. Moreover, angioleiomyomas and myopericytomas are now classified in the latest WHO classification, and fall within the same morphological spectrum of the perivascular tumor type [83]. Our dural cases present clinical (affecting adults between the fourth and the sixth decades) [82], and radiological similarities with soft tissue angioleiomyomas (such as hyperintensity on T2-weighted imaging and a strong enhancement after contrast injection) [84]. Furthermore, we showed that the same recurrent substitution (*GJA4* p.Gly41Cys) was shared by soft tissue and intracranial ALM, reinforcing their close vicinity. This mutation was probably not described before, due to the lack of comprehensive molecular studies of this frequent (in soft tissue) and benign tumor. Here, we did not find any copy number variations, contrary to other previous soft tissue studies (showing cytogenetic abnormalities including monsonosmy of chromosome 13, and loss of 6p, 21q, and 13q), which however, are lacking a histopathological characterization [85–87]. Based on epigenetic profiling, all intracranial tumors clearly clustered together but were different from all other established groups, particularly soft tissue angioleiomyomas and meningiomas. Since DNA methylation profiles are thought to represent a combination of both somatically acquired DNA methylation changes and a signature reflecting the cell of origin [88], it is reasonable to assume that tumors from the dura mater represent a distinct tumor type from soft tissue angioleiomyomas. Based on our center’s experience, the prevalence of DALMs seems to be low, representing only 1.5% (3/202 cases) of CNS vascular and perivascular lesions resected over the past 20 years. However, this tumor type is probably misdiagnosed, and variably called “angioleiomyoma”, “myopericytoma”, “venous hemangioma” and “cavernous hemangioma”. Moreover, the terminology of “angioleiomyomas” is probably not widely known and consequently rarely used by neuropathologists. Finally, because the natural history of DALM seems very favourable, a subset of patients is probably not resected, these lesions being radiologically interpreted as meningiomas.

In summary, we performed for the first time a comprehensive analysis of a distinctive intradural perivascular tumor type presenting histopathological similarities with soft tissue angioleiomyomas, frequently having *GJA4* mutations. Because of its dural location and distinct DNA methylation profile, we suggest the term “dural angioleiomyoma” for this benign tumor.

## References

[1] Ganz JC (2022). Intracranial arteriovenous malformations. Prog Brain Res.

[2] Venugopal V, Sumi S (2022). Molecular biomarkers and drug targets in brain arteriovenous and cavernous malformations: Where are we?. Stroke.

[3] Yang Z, Yu G, Zhu W, Chen L, Song J, Mao Y (2021). The benefit and outcome prediction of acute surgery for hemorrhagic brainstem cavernous malformation with impending respiratory failure. J Clin Neurosci Off J Neurosurg Soc Australas.

[4] Wu C-X, Zang Z-X, Hong T, Dong M-Q, Shan Y, Zhao Z-L (2021). Signal intensity ratio of draining vein on silent MR angiography as an indicator of high-flow arteriovenous shunt in brain arteriovenous malformation. Eur Radiol.

[5] Sabayan B, Lineback C, Viswanathan A, Leslie-Mazwi TM, Shaibani A (2021). Central nervous system vascular malformations: a clinical review. Ann Clin Transl Neurol.

[6] Dasgupta R, Fishman SJ (2014). ISSVA classification. Semin Pediatr Surg.

[7] Wassef M, Blei F, Adams D, Alomari A, Baselga E, Berenstein A (2015). Vascular anomalies classification: recommendations from the international society for the study of vascular anomalies. Pediatrics.

[8] McCormick WF, Boulter TR (1966). Vascular malformations (“angiomas”) of the dura mater. J Neurosurg.

[9] Barrow DL (1997). Classification and natural history of cerebral vascular malformations: arteriovenous, cavernous, and venous. J Stroke Cerebrovasc Dis Off J Natl Stroke Assoc.

[10] Challa VR, Moody DM, Brown WR (1995). Vascular malformations of the central nervous system. J Neuropathol Exp Neurol.

[11] Awad IA, Robinson JR, Mohanty S, Estes ML (1993). Mixed vascular malformations of the brain: clinical and pathogenetic considerations. Neurosurgery.

[12] Gaztanaga W, Luther E, McCarthy D, Chamyan G, Wang S, Ragheb J (2021). Giant, symptomatic mixed vascular malformation containing a cavernoma, developmental venous anomaly, and capillary telangiectasia in a 19-month-old infant. Childs Nerv Syst ChNS Off J Int Soc Pediatr Neurosurg.

[13] Bergametti F, Denier C, Labauge P, Arnoult M, Boetto S, Clanet M (2005). Mutations within the programmed cell death 10 gene cause cerebral cavernous malformations. Am J Hum Genet.

[14] Denier C, Labauge P, Bergametti F, Marchelli F, Riant F, Arnoult M (2006). Genotype-phenotype correlations in cerebral cavernous malformations patients. Ann Neurol.

[15] Hong T, Xiao X, Ren J, Cui B, Zong Y, Zou J (2021). Somatic MAP3K3 and PIK3CA mutations in sporadic cerebral and spinal cord cavernous malformations. Brain J Neurol.

[16] Labauge P, Denier C, Bergametti F, Tournier-Lasserve E (2007). Genetics of cavernous angiomas. Lancet Neurol.

[17] Laberge-le Couteulx S, Jung HH, Labauge P, Houtteville JP, Lescoat C, Cecillon M (1999). Truncating mutations in CCM1, encoding KRIT1, cause hereditary cavernous angiomas. Nat Genet.

[18] Liquori CL, Berg MJ, Siegel AM, Huang E, Zawistowski JS, Stoffer T (2003). Mutations in a gene encoding a novel protein containing a phosphotyrosine-binding domain cause type 2 cerebral cavernous malformations. Am J Hum Genet.

[19] Weng J, Yang Y, Song D, Huo R, Li H, Chen Y (2021). Somatic MAP3K3 mutation defines a subclass of cerebral cavernous malformation. Am J Hum Genet.

[20] Nikolaev SI, Vetiska S, Bonilla X, Boudreau E, Jauhiainen S, Rezai Jahromi B (2018). Somatic activating KRAS mutations in arteriovenous malformations of the brain. N Engl J Med.

[21] Capper D, Jones DTW, Sill M, Hovestadt V, Schrimpf D, Sturm D (2018). DNA methylation-based classification of central nervous system tumours. Nature.

[22] Koelsche C, Schrimpf D, Stichel D, Sill M, Sahm F, Reuss DE (2021). Sarcoma classification by DNA methylation profiling. Nat Commun.

[23] Louis DN, Perry A, Wesseling P, Brat DJ, Cree IA, Figarella-Branger D (2021). The 2021 WHO classification of tumors of the central nervous system: a summary. Neuro-Oncol.

[24] Colnat-Coulbois S, Schmitt E, Klein O, Weinbreck N, Auque J, Civit T (2008). Angioleiomyoma of the cavernous sinus: case report. Neurosurgery.

[25] Altieri R, Morrone A, Certo F, Parisi G, Buscema G, Broggi G (2019). Tentorial angioleiomyoma: a rare neurosurgical entity. Case report and literature review. World Neurosurg.

[26] Biondi A, Clemenceau S, Dormont D, Deladoeuille M, Ricciardi GK, Mokhtari K (2002). Intracranial extra-axial cavernous (HEM) angiomas: tumors or vascular malformations?. J Neuroradiol J Neuroradiol.

[27] Boockvar JA, Stiefel M, Malhotra N, Dolinskas C, Dwyer-Joyce C, LeRoux PD (2005). Dural cavernous angioma of the posterior sagittal sinus: case report. Surg Neurol.

[28] Bteich F, Kassab C, Hage GE, Moussa R, Abadjian GA, Nassif RB (2019). Atypical presentation of a parietal convexity dural-based cavernous hemangioma: a case report and review of the literature. World Neurosurg.

[29] Calle S, Louis D, Westmark R, Westmark K (2016). Angioleiomyoma of the falx. J Radiol Case Rep.

[30] Conner TM, Waziri A, Kleinschmidt-Demasters BK (2012). Angioleiomyomas of the dura: rare entities that lack KRIT1 mutations. Am J Surg Pathol.

[31] Cruz AS, Jeyamohan S, Moisi M, Tubbs RS, Page J, Chamiraju P (2016). Dural-based cavernoma of the posterior cranial fossa mimicking a meningioma: a case report. Cureus.

[32] Di Vitantonio H, De Paulis D, Ricci A, Marzi S, Dehcordi SR, Galzio RJ (2015). Cavernous hemangioma of the dura mater mimicking meningioma. Surg Neurol Int.

[33] Dörner L, Buhl R, Hugo HH, Jansen O, Barth H, Mehdorn HM (2005). Unusual locations for cavernous hemangiomas: report of two cases and review of the literature. Acta Neurochir (Wien).

[34] Figueiredo EG, Gomes M, Vellutini E, Rosemberg S, Marino R (2005). Angioleiomyoma of the cavernous sinus: case report. Neurosurgery.

[35] Fraser JF, Mass AY, Brown S, Anand VK, Schwartz TH (2008). Transnasal endoscopic resection of a cavernous sinus hemangioma: technical note and review of the literature. Skull Base Off J North Am Skull Base Soc Al.

[36] Gasco J, Franklin B, Rangel-Castilla L, Campbell GA, Eltorky M, Salinas P (2009). Infratentorial angioleiomyoma: a new location for a rare neoplastic entity. J Neurosurg.

[37] Ghanta RK, Tangella P, Koti K, Dandamudi S (2013). A rare case of an extra-axial cavernous angioma in the cerebellopontine angle. J Neurosci Rural Pract.

[38] Gravbrot N, Brasiliense LBC, Alswied A, Sun B, Lemole GM (2019). Multiple extraaxial cavernous hemangiomas: rare entity. World Neurosurg.

[39] Gupta RK, Saran RK, Jagetia A, Narang P (2016). Extra-axial dural cavernous hemangioma with dural tail sign, masquerading as meningioma. J Neurosci Rural Pract.

[40] Gutiérrez-González R, Casanova-Peño I, Porta-Etessam J, Martínez A, Boto GR (2010). Dural cavernous haemangioma of the anterior cranial fossa. J Clin Neurosci Off J Neurosurg Soc Australas.

[41] Hashimoto M, Yokota A, Ohta H, Urasaki E (2000). Intratumoral injection of plastic adhesive material for removal of cavernous sinus hemangioma. Tech Note J Neurosurg.

[42] He K, Chen L, Zhu W, Cheng H, Wang Y, Mao Y (2014). Diagnosis and surgical treatment of cavernous sinus angioleiomyoma: a report of four cases. Jpn J Clin Oncol.

[43] Hirano H, Tashiro Y, Fujio S, Goto M, Arita K (2011). Diffuse large B-cell lymphoma within a cavernous hemangioma of the cavernous sinus. Brain Tumor Pathol.

[44] Hwang SW, Pfannl RM, Wu JK (2009). Convexity dural cavernous malformation with intradural and extradural extension mimicking a meningioma: a case report. Acta Neurochir (Wien).

[45] Wang X, Liu J-P, You C, Mao Q (2016). Convexity dural cavernous haemangioma mimicking meningioma: a case report. Br J Neurosurg.

[46] Ito M, Kamiyama H, Nakamura T, Nakajima H, Tokugawa J (2009). Dural cavernous hemangioma of the cerebellar falx. Neurol Med Chir (Tokyo).

[47] Kanaan I, Jallu A, Alwatban J, Patay Z, Hessler R (2001). Extra-axial cavernous hemangioma: two case reports. Skull Base Off J North Am Skull Base Soc Al.

[48] Katayama Y, Tsubokawa T, Miyazaki S, Yoshida K, Himi K (1991). Magnetic resonance imaging of cavernous sinus cavernous hemangiomas. Neuroradiology.

[49] Khattar NK, Adams SW, Schaber AS, White AC, Al Ghamdi M, Hruska RT (2018). Endoscopic endonasal surgery for the resection of a cavernous hemangioma with a sellar extension. Cureus.

[50] Lee AG, Parrish RG, Goodman JC (1998). Homonymous hemianopsia due to a dural cavernous hemangioma. J Neuro-Ophthalmol Off J North Am Neuro-Ophthalmol Soc.

[51] Li C-B, Xie M-G, Ma J-P, Wang L, Hao S-Y, Zhang L-W (2018). Primary intracranial angioleiomyomas as rare, nonmalignant, and distinct neoplastic entities: a series of 8 cases and a literature review. World Neurosurg.

[52] Li D, Hao S-Y, Tang J, Cao X-Y, Lin S, Wang J-M (2014). Primary intracranial angioleiomyomas: diagnosis, treatment, and literature review. Brain Tumor Pathol.

[53] Mansour TR, Medhkour Y, Entezami P, Mrak R, Schroeder J, Medhkour A (2017). The art of mimicry: anterior clinoid dural-based cavernous hemangioma mistaken for a meningioma. World Neurosurg.

[54] McKechnie S, Harper C, Besser M (1998). Durally-based occipital cavernous haemangioma indistinguishable from meningioma. J Clin Neurosci Off J Neurosurg Soc Australas.

[55] Meincke J, Lützen N, Doostkam S, Urbach H (2018). Teaching neuroimages: “filling out” in cavernous hemangioma of the cavernous sinus. Clin Neuroradiol.

[56] Melone AG, Delfinis CP, Passacantilli E, Lenzi J, Santoro A (2010). Intracranial extra-axial cavernous angioma of the cerebellar falx. World Neurosurg.

[57] Ohata K, El-Naggar A, Takami T, Morino M, El-Adawy Y, El-Sheik K (1999). Efficacy of induced hypotension in the surgical treatment of large cavernous sinus cavernomas. J Neurosurg.

[58] Oya S, Prayson RA, Lee JH (2012). A tentorial venous hemangioma presenting as an extra-axial mass in the ambient cistern: a case report. J Neurol Surg Rep.

[59] Puca A, Colosimo C, Tirpakova B, Lauriola L, Di Rocco F (2004). Cavernous hemangioma extending to extracranial, intracranial, and orbital regions. Case Rep J Neurosurg.

[60] Revuelta R, Teixeira F, Rojas R, Juambelz P, Romero V, Valdes J (1994). Cavernous hemangiomas of the dura mater at the convexity. Report of a case and therapeutical considerations. Neurosurg Rev.

[61] Rushton AW, Ng HK, Metreweli C (1999). Dural cavernous haemangioma with bony infiltration. Clin Radiol.

[62] Sakakibara Y, Matsumori T, Taguchi Y, Koizumi H (2010). Supratentorial high convexity intradural extramedullary cavernous angioma: case report. Neurol Med Chir (Tokyo).

[63] Sathi S, Folkerth R, Madsen JR (1992). Cavernous angioma of the posterior fossa dura mimicking a meningioma: case report and review of literature. Surg Neurol.

[64] Seo Y, Fukuoka S, Sasaki T, Takanashi M, Hojo A, Nakamura H (2000). Cavernous sinus hemangioma treated with gamma knife radiosurgery: usefulness of SPECT for diagnosis–case report. Neurol Med Chir (Tokyo).

[65] Sun L, Zhu Y, Wang H (2014). Angioleiomyoma, a rare intracranial tumor: 3 case report and a literature review. World J Surg Oncol.

[66] Tannouri F, Divano L, Caucheteur V, Hacourt A, Pirotte B, Salmon I (2001). Cavernous haemangioma in the cavernous sinus: case report and review of the literature. Neuroradiology.

[67] Teranishi Y, Kohno M, Sora S, Sato H, Yokoyama M (2014). Cavernous sinus angioleiomyoma: case report and review of the literature. J Neurol Surg Rep.

[68] Tsutsumi S, Yasumoto Y, Saeki H, Ito M (2014). Cranial dural cavernous angioma. Clin Neuroradiol.

[69] Xiaofeng L, Hongzhi X, Junrui C (2016). Primary angioleiomyoma in the cavernous sinus: a case report. World Neurosurg.

[70] Zeng X, Mahta A, Kim RY, Saad AG, Kesari S (2012). Refractory seizures due to a dural-based cavernoma masquerading as a meningioma. Neurol Sci Off J Ital Neurol Soc Ital Soc Clin Neurophysiol.

[71] Zhang CH, Hasegawa H, Johns P, Martin AJ (2015). Myopericytoma of the posterior cranial fossa. Br J Neurosurg.

[72] Reed KE, Westphale EM, Larson DM, Wang HZ, Veenstra RD, Beyer EC (1993). Molecular cloning and functional expression of human connexin37, an endothelial cell gap junction protein. J Clin Invest.

[73] Simon AM, McWhorter AR (2002). Vascular abnormalities in mice lacking the endothelial gap junction proteins connexin37 and connexin40. Dev Biol.

[74] Ugwu N, Atzmony L, Ellis KT, Panse G, Jain D, Ko CJ (2021). Cutaneous and hepatic vascular lesions due to a recurrent somatic GJA4 mutation reveal a pathway for vascular malformation. HGG Adv.

[75] Tittarelli A, Guerrero I, Tempio F, Gleisner MA, Avalos I, Sabanegh S (2015). Overexpression of connexin 43 reduces melanoma proliferative and metastatic capacity. Br J Cancer.

[76] Aasen T, Mesnil M, Naus CC, Lampe PD, Laird DW (2016). Gap junctions and cancer: communicating for 50 years. Nat Rev Cancer.

[77] Abbruzzese C, Mattarocci S, Pizzuti L, Mileo AM, Visca P, Antoniani B (2012). Determination of SGK1 mRNA in non-small cell lung cancer samples underlines high expression in squamous cell carcinomas. J Exp Clin Cancer Res CR.

[78] Talarico C, Dattilo V, D’Antona L, Barone A, Amodio N, Belviso S (2016). SI113, a SGK1 inhibitor, potentiates the effects of radiotherapy, modulates the response to oxidative stress and induces cytotoxic autophagy in human glioblastoma multiforme cells. Oncotarget.

[79] Chung EJ, Sung YK, Farooq M, Kim Y, Im S, Tak WY (2002). Gene expression profile analysis in human hepatocellular carcinoma by cDNA microarray. Mol Cells.

[80] Catela C, Kratsios P, Hede M, Lang F, Rosenthal N (2010). Serum and glucocorticoid-inducible kinase 1 (SGK1) is necessary for vascular remodeling during angiogenesis. Dev Dyn Off Publ Am Assoc Anat.

[81] Hachisuga T, Hashimoto H, Enjoji M (1984). Angioleiomyoma. A clinicopathologic reappraisal of 562 cases. Cancer.

[82] Matsuyama A, Hisaoka M, Hashimoto H (2007). Angioleiomyoma: a clinicopathologic and immunohistochemical reappraisal with special reference to the correlation with myopericytoma. Hum Pathol.

[83] Sbaraglia M, Bellan E, Dei Tos AP (2021). The 2020 WHO classification of soft tissue tumours: news and perspectives. Pathologica.

[84] Yoo HJ, Choi J-A, Chung J-H, Oh JH, Lee G-K, Choi J-Y (2009). Angioleiomyoma in soft tissue of extremities: MRI findings. AJR Am J Roentgenol.

[85] Nilbert M, Mandahl N, Heim S, Rydholm A, Willén H, Mitelman F (1989). Cytogenetic abnormalities in an angioleiomyoma. Cancer Genet Cytogenet.

[86] Nishio J, Iwasaki H, Ohjimi Y, Ishiguro M, Kobayashi K, Nabeshima K (2004). Chromosomal imbalances in angioleiomyomas by comparative genomic hybridization. Int J Mol Med.

[87] Welborn J, Fenner S, Parks R (2010). Angioleiomyoma: a benign tumor with karyotypic aberrations. Cancer Genet Cytogenet.

[88] Hovestadt V, Jones DTW, Picelli S, Wang W, Kool M, Northcott PA (2014). Decoding the regulatory landscape of medulloblastoma using DNA methylation sequencing. Nature.

